# Basic and translational aging research in China: present and future

**DOI:** 10.1007/s13238-019-0617-0

**Published:** 2019-04-02

**Authors:** Xiaojuan He, Moshi Song, Jing Qu, Yansu Guo, Heqi Cao, Ruijuan Sun, Guang-Hui Liu, Yong Shen, Major Program Expert Group

**Affiliations:** 1 Advanced Innovation Center for Human Brain Protection, National Clinical Research Center for Geriatric Disorders, Xuanwu Hospital, Capital Medical University, Beijing, 100053 China; 2 National Laboratory of Biomacromolecules, CAS Center for Excellence in Biomacromolecules, Institute of Biophysics, Chinese Academy of Sciences, Beijing, 100101 China; 3 State Key Laboratory of Membrane Biology, Institute of Zoology, Chinese Academy of Sciences, Beijing, 100101 China; 4 University of Chinese Academy of Sciences, Beijing, 100049 China; 5 Institute for Stem Cell and Regeneration, Chinese Academy of Sciences, Beijing, 100101 China; 6 State Key Laboratory of Stem Cell and Reproductive Biology, Institute of Zoology, Chinese Academy of Sciences, Beijing, 100101 China; 7 Department of Health Sciences, National Natural Science Foundation of China, Beijing, 100085 China; 8 University of Science and Technology of China, Hefei, 230027 China

**The percentage of elderly people in the world is increasing at an unprecedented pace; so it is in China, which has the world’s largest population and a high ratio of the seniors (aged 60 and above) to working-age adults. The growing elderly population is presenting a major social challenge. Accordingly, it is not only imperative as a national strategic demand but also promises great scientific values to understand the biological process of aging, explore the mystery of healthy aging, delay the aging process, and treat the age-related diseases. This Perspective summarizes past and present advances of the basic and translational aging research in China and offers perspectives on future endeavors in this area.**

Based on a national survey conducted by the National Bureau of Statistics, China’s elderly population (aged 60 and above) had reached 249 million by the end of 2018, making up about 17.9% of the total population; those aged 65 and above totaled 167 million, accounting for 11.9% of the total population. The 2013 “China Aging Development Report” pointed out that the severity of the aging problems in China was unprecedented. Population aging is accompanied by increased prevalence of various age-related chronic diseases. The cost of healthcare for the elderlies is imposing a heavy financial burden both on the elderlies and their families as well as on society as a whole. It is of great social significance to achieve healthy aging to reduce medical expenses and increase productive engagement of elderly population.

The history of aging research in China can be traced back to early 1980s, Chinese scientists began to decode aging. Prof. Tanjun Tong’s team discovered the relationship between P16 and telomeres, unraveled the molecular mechanisms by which P16 regulates cellular senescence, and identified genetic indicators and quantitative indices for estimating the “age” of human cells, including telomere length, cell proliferative capacity, senescence-associated β-galactosidase activity, advanced glycation end products, DNA damage repair ability, DNA methylation degree, mitochondrial DNA deletion, and α-2-macroglobulin protein level (Zeng et al., 1999; Duan et al., 2001; Wang et al., 2001; Zhang et al., 2003). In 2005, Liu et al. discovered that a mutation in Lamin A impairs DNA damage repair, thus destabilizing the genome and causing premature cellular aging (Liu et al., 2005). Liu et al. further discovered that Lamin A regulates stem cell self-renewal via the stimulation of the longevity protein SIRT1 (Liu et al., 2012a). Since then, Chinese scientists have been elucidating molecular mechanism, screening drug candidates, and conducting clinical research in the area of aging and degeneration, continuously making progress and filling gaps in the field of aging and degeneration.

**Figure 1:**
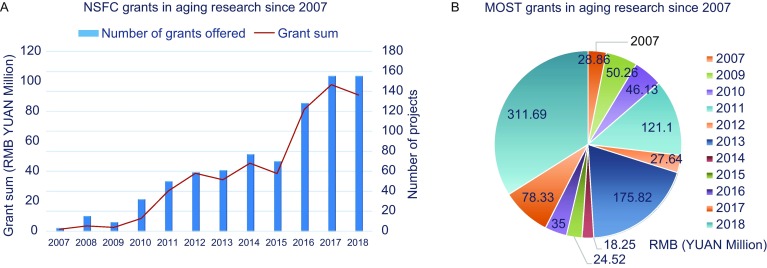
**Chinese government grant supports to aging research since 2007**. (A) NSFC grant supports to aging research. The number of funded projects increased from only a few in 2007 to 156 in 2018; the total amount of grant money increased from several to 90 million RMB Yuan. (B) MOST grant supports to aging research. A total of 37 projects were supported since 2007 with a total grant money of 917.60 million RMB Yuan

Over the past ten years, the Chinese government has focused on basic and translational aging research by establishing a number of laboratories, research centers and institutions for aging research and providing significant financial support. The National Natural Science Foundation of China (NSFC) and the Ministry of Science and Technology of China (MOST) supported numerous projects with grants totaling 470.18 million and 917.60 million RMB Yuan from 2007 to 2018, respectively (Fig. 1).

China has made remarkable progress in aging research that has garnered international recognition over the past decade. In 2016, China launched the Major Program on Organ Aging and Degeneration. Organ aging is accompanied by the progressive decline in organ function. At the cellular level, aging is characterized by the presence of an array of hallmarks, including genomic instability, telomere attrition, epigenetic alterations, loss of proteostasis, deregulated nutrient sensing, mitochondrial dysfunction, cellular senescence, stem cell exhaustion, and altered intercellular communication (López-Otín et al., 2013). Further progression of organ degeneration leads to the development of age-related diseases. Accordingly, the prognosis and prevention of organ degeneration is critical to the prevention of aging-associated chronic diseases and the primary goal of the Major Program on Organ Aging and Degeneration.

The Major Program on Organ Aging and Degeneration aims to answer the following scientific questions: 1) What are the molecular, cellular, and functional changes during aging and progressive degeneration of different organs? 2) How do genetic and environmental factors affect organ aging and degeneration? 3) What are the regulatory mechanisms for organ aging and degeneration during the development of age-related diseases? Towards these questions, the Chinese aging research community have been working collectively with distinct advantages including abundant clinical resources and newly developed technologies. In China, aging biobanks including centenarian collections and brain samples from AD patients have gradually taken shape. Blood samples from 2,178 centenarians and their offsprings have been collected for genetic analysis of a series of age-related diseases, laying the foundations for studying mechanisms and identifying biomarkers for aging and age-related diseases (Zeng et al., 2016). New technologies have also been developed and employed, such as bio-imaging, multi-level “omics” analysis, systems biology modeling and simulation, tissue-specific genetic interventions, stem cell techniques, tissue reconstruction, and premature aging and longevity animal model generation, facilitating the unraveling of molecular mechanisms and therapeutic targets of organ aging and degeneration at the molecular, cellular, tissue, and organism levels.

Since its launch in 2016, the Major Program on Organ Aging and Degeneration has greatly promoted the basic and translational aging research in China. The following is a summary of some recent Made-in-China research progress in four aspects.

The first is the progress made in genetic and epigenetic regulation of organ aging and degeneration. Aging is a complex biological process. The progression from organ aging to degeneration is collectively influenced by genetic and epigenetic factors. Identification of key tissue-specific aging-related genes, RNAs, proteins, and other biomacromolecules are essential to the mechanistic studies and intervention of human organ aging, as well as to the prognosis and diagnosis of age-related diseases.

Limited by insufficient sample size of centenarians, only two genome-wide significant loci, TOMM40/APOE/APOC1 and chromosome 5q33.3, have been found to associate with longevity. Zeng et al. reported a total 11 independent sites associated with longevity in a genome-wide association study (GWAS) on 2,178 Han Chinese centenarians, which is a sample size 1.7 times larger than the previous largest GWAS on centenarians (Sebastiani et al., 2012). Eight independent SNPs and the two aforementioned loci overlapped across the Han Chinese, European, and US populations (Zeng et al., 2016).

Approximately 90% of the genome is transcriptionally active, of which only 1.5% encodes proteins. The rest encodes non-protein products such as long-chain non-coding RNAs (lncRNAs) and small ncRNAs including microRNAs (miRNAs), P-element-induced wimpy testis-interacting RNAs (piRNAs), and small interfering RNAs (siRNAs) (Yang et al., 2014; Awan et al., 2017). The majority of lncRNAs are developmental stage-, tissue-, and cell-type-specific. Liu et al. reported 18 lncRNA as well as 14 mRNA modules anatomically diversified by spatial, age, and sex specificities in rhesus macaque brains and characterized the dynamic changes in lncRNA expression during brain development and aging (Liu et al., 2017).

Repetitive sequence-enriched genomic regions such as centromeres, telomeres, and most heterochromatin regions are transcriptionally inactive and may even impede replication fork progression, causing replicative stress and disrupting genomic integrity that may lead to aging and tumorigenesis. Mendez-Bermudez et al. reported that telomere-specific protective protein TRF2 stabilizes telomere and peri-centromere regions and is critical to the maintenance of chromosome stability, emphasizing the interconnection between heterochromatin replication and aging (Mendez-Bermudez et al., 2018). Deng et al. revealed that TOE1 interacts with and mediates the 3′ processing of hTR as a 3′-to-5′ exonuclease in conjunction with PARN whereas in TOE1-deficient cells hTR precursors are enriched along with decreased telomerase activity and telomere shortening, elucidating a mechanistic association between TOE1 mutation, abnormal hTR processing, and telomere dysfunction. Interestingly, mutations of PARN and TOE1 have been implicated in human diseases with premature aging phenotypes (Deng et al., 2018).

Telomere shortening during cell replication is a major cause of replicative senescence and intensively studied during organ aging and degeneration. Telomeres and telomere-binding proteins form complex secondary nucleoprotein structures that are critical for genome integrity but also present serious challenges during telomere DNA replication. Li et al. reported that the mitotic checkpoint protein BUB3 can bind to telomeres while BUB1 facilitates the recruitment of helicase BLM for telomere DNA replication via the phosphorylation of telomere-binding protein TRF1 (Li et al., 2018). Liu et al. discovered a new telomerase-binding protein ribosomal maturation factor (SBDS) that colocalizes with telomeres and binds to TPP1 as a stabilizer for TPP1-telomerase interaction during DNA replication so as to maintain telomere length, providing a plausible explanation of how SBDS mutations accelerate telomere-length shortening in over 90% of the Shwachman-Diamond Syndrome (SDS) patients (Liu et al., 2018a, b). Tang et al. found that the RNA-binding protein HuR binds to TREC and promotes the methylation (m5C) of TREC at C106, thus enhancing telomerase activity and maintaining the self-renewal ability of murine hematopoietic stem cells. By contrast, in patients with Dyskeratosis congenital (DC) TREC-U100A mutant that interrupts the interaction with HuR impairs telomerase activity and causes shortened telomere, associating HuR with telomerase activity and TERC-linked DC (Tang et al., 2018).

Epigenetics of aging provides deep analysis of the epigenetic status of aging, including nucleic acid and histone modifications. In mouse and human brains, Cheng et al. revealed distinct H3K27ac modification patterns in genes differentially regulated with age, and overactivated inflammation-related genes are marked by decreased broad gene-body hyperacetylation (Cheng et al., 2018). Wang et al. reported that hematopoietic stem cells (HSCs) require the histone deacetylase SIRT6 for maintaining homeostasis via the regulation of Wnt signaling (Wang et al., 2016a). Zhang et al. used gene-editing technologies and created genetically engineered SIRT6-deficient monkeys. These monkeys have significantly increased H3K56ac and birth defects as SIRT6 directs neural stem cell differentiation as well as brain development by regulating the expression of imprinting lncRNA H19 (Zhang et al., 2018b). Wang et al. revealed that YTHDF2 facilitates the decay of m6A-modified mRNAs of Wnt target genes in hematopoietic stem cells (HSCs), contributing to the repression of Wnt signaling at steady state. Under hematological stresses, YTHDF2 deficiency blocks the degradation of mRNAs of both Wnt target genes and survival-related genes and increases the number and regenerative capacity of HSCs (Wang et al., 2018a, b).

Genetic mutations are associated with the development of accelerated aging and aging-related disorders. Notably, mutations in *LMNA* and *WRN* genes lead to aberrant splicing product progerin and protein loss in human premature aging disorders Hutchinson-Gilford progeria syndrome (HGPS) and Werner syndrome (WS), respectively (Kudlow et al., 2007; Liu et al., 2011a, b; Zhang et al., 2015). Studies on how genetic alteration leads to the cellular and organismal phenotypes of premature aging provide clues to the molecular mechanisms underlying physiological aging and facilitate our understanding of the molecular pathways contributing to healthy aging. Liu’s group has generated induced pluripotent stem cells (iPSCs) from fibroblasts obtained from patients with HGPS, Parkinson’s disease (PD), Amyotrophic lateral sclerosis (ALS), Fanconi Anemia (FA), and Xeroderma pigmentosum (XP) (Liu et al., 2011a, b, 2012b, 2014a; Fu et al., 2016; Wang et al., 2017). Upon the differentiation of these disease-specific iPSCs to specific somatic cell types, the latter recapitulated aging/disease-associated and tissue-specific phenotypic defects. Furthermore, using targeted gene-editing techniques, they have successfully corrected the mutated *LMNA* in HGPS-iPSCs, mutated *LRRK2* in PD-iPSCs, mutated *FANCA* in FA-iPSCs, as well as mutated *SOD1* and *FUS* in ALS-iPSCs (Liu et al., 2011b, 2012b, 2014a; Kubben et al., 2016; Wang et al., 2017). These stem cell-based studies provide important platforms for studying aging/disease mechanisms and developing new therapies (Geng et al., 2018; Wu et al., 2018; Zhang et al., 2018a; Ling et al., 2019).

The second aspect is how cellular homeostasis deregulated in organ aging and degeneration. Human organ aging and degeneration are accompanied by the changes in the intracellular and extracellular environments, key metabolic signaling pathways, and cellular senescence induced by free radicals, hormones, and proinflammatory cytokines.

Bone diseases, such as osteoarthritis, disc herniation, and osteoporosis, are often related to changes in the intracellular homeostasis of aging organs. Xu et al. found that FOXP2 regulates bone remodeling and controls bone formation in cooperation with FOXP1 and RBPjk by forming a complex regulating Notch signaling pathway (Xu et al., 2018c). Osteoporosis is related to dyshomeostatic energy metabolism and antioxidant system. Gao et al. observed that the activation of manganese superoxide dismutase (SOD2) occurs along with decreased mitochondrial oxidative stress during osteoblast differentiation and revealed that the histone deacetylase sirtuin 3 (SIRT3) enhances the antioxidant ability of osteoblasts via SOD2 deacetylation and thus maintains mitochondrial stability, indicative of the therapeutic potential of SIRT3 in the prevention and treatment of metabolism-related bone diseases (Gao et al., 2018b).

Hypogonadism often occurs in middle-aged and older men. Gao et al. found that in testicular Leydig cells impaired autophagy associated with decreased testosterone levels interrupts cholesterol absorption and androgen synthesis, indicating the causative link between impaired autophagy and hypogonadism (Gao et al., 2018a).

Cellular senescence and tumor development and progression are often linked to the changes in the activities of cell cycle regulators. p63 is a member of the p53 transcription factor family and participates in cell cycle regulation and development of certain tumors. Chen et al. elucidated a new regulatory pathway in cellular aging: ∆Np63α promotes the transcription of E3 ubiquitin ligase, HERC3, which subsequently mediates the ubiquitination and degradation of MM1 and de-represses c-Myc, thereby alleviating cellular senescence (Chen et al., 2018). Chronic inflammation is implicated in age-related diseases. In senescent cells, DNA that leaks from the nucleus activates the cGAS-STING pathway and triggers innate immune. Liu et al. found that DNA damage promotes the nuclear translocation of cGAS in human cell and mouse models. Upon entry into the nucleus, cGAS interacts with PARP1 and inhibits the formation of PARP1-Timeless complex, thereby suppressing homologous recombination-mediated DNA repair and promoting tumorigenesis, indicative of cGAS as a novel therapeutic target for cancer (Liu et al., 2018a).

Centenarians are ideal models to study human longevity and healthy aging. Wang et al. showed that the autophagy-lysosomal pathway is significantly upregulated in centenarians based on a study using the transcripts from 76 centenarians. The overexpression of genes such as *CTSB*, *ATP6V0C*, *ATG4D* and *WIPI1* of this pathway promotes autophagy and delays senescence in cultured IMR-90 cells, while the overexpression of the homologs of *WIPI1* and *Atg18a* extends lifespan in Drosophila, indicating that the enhancement of autophagy activities may be an important and conserved mechanism in prolonging lifespan (Wang et al., 2018b).

Increasing evidence has emerged to support the relationship between metabolic disorders and age-related diseases. Wang et al. reported an important role of miR-152 in the positive regulation of hepatic glycogen production (Wang et al., 2016c) and Liu et al. showed that growth factor receptor binding protein 10 (GRB10) promotes lipid breakdown and heat production by negative regulation of mTORC1 (Liu et al., 2014b).

Huntington’s disease is an autosomal-dominant neurodegenerative disease mainly caused by accumulation of cytotoxic HTT protein (mHTT), which disrupts protein homeostasis. Yu et al. found that two kinases HIPK3 and MAPK11 negatively regulates autophagy, thereby resulting in the excessive accumulation of mHTT protein in Huntington’s disease (HD) cells. Interestingly, the regulation of mHTT by MAPK11 and HIPK3 is mHTT-dependent, which provides a feedback mechanism by which mHTT enhances its own level that contributes to mHTT accumulation and disease progression. Consistently, knockdown of MAPK11 and HIPK3 significantly alleviates disease-related behavioral phenotypes in HD mice (Yu et al., 2017).

The third is to identifying novel biomarkers for organ aging and degeneration. Molecular biomarkers are widely used in the prognosis and diagnosis of diseases. In the research of age-related diseases, biomarkers provide a dynamic and powerful approach to understanding the pathogenesis of diseases but have also enabled early clinical diagnosis.

Gan et al. compared the levels of nucleic acid oxidation and metabolites in blood and urine of mice, rats and rhesus monkeys, and showed that the levels of RNA oxidative metabolites including 8-oxoguanosine (8-OG) in urine significantly increase with age, pointing out urine 8-OG as a novel potential biomarker for age estimation in mammals and humans (Gan et al., 2018).

Incidences of neurodegenerative diseases, including Alzheimer’s disease (AD), Amyotrophic lateral sclerosis (ALS), Huntington’s disease (HD), Parkinson’s disease (PD), and frontotemporal dementia (FTD), are frequently positively correlated with age. Given the current lack of cure for these neurodegenerative diseases, early diagnosis by using molecular biomarkers is of clinical relevance to slowing disease progression. Cheng et al. reported increased serum level and activity of beta-secretase (BACE1) in patients with mild cognitive impairment (MCI) and AD that are well correlated with clinical severity, thus likely applicable to the prediction of AD at early stages (Cheng et al., 2014).

Partial loss of TBK1 function is a major genetic cause for multiple age-related neurodegenerative diseases including ALS and FTD, but the underlying mechanism was unclear. Xu et al. reported that TBK1 acts as an endogenous suppressor of RIPK1. In TBK1-deficient population, the decrease of TAK1 with age further promotes the activation of RIPK1 that leads to ALS and FTD. In mice, inhibition of RIPK1 activity effectively reverses disease symptoms and behavioral changes, suggesting that over-activation of RIPK1 is a key factor in the pathogenesis of and a potential therapeutic target against ALS and FTD (Xu et al., 2018b).

UV exposure is a major factor for skin aging. Yi et al reported that UV irradiation reduces the expression of DNA methyltransferase 1 (DNMT1) and *p53* promoter methylation, thus increasing P53 expression and promoting cellular senescence (Yi et al., 2018). Xie et al. revealed that miR-377 induces senescence in human skin fibroblasts as a negative regulator of DNMT1, highlighting a novel role for miR-377-DNMT1-p53 axis in skin aging (Xie et al., 2017).

The last is to developing new technologies for studying organ aging and degeneration. Supported by the Major Program on Organ Aging and Degeneration, a series of new tools and technologies have been developed by the scientists in China aging research community to better solve major challenges on organ aging and degeneration.

Over the past decade, the technology for visualizing small-molecule metabolites has greatly improved. By rational design and proper modification of substrate-binding proteins, Zhao et al. developed a series of genetically encoded high-performance fluorescent indicators for NADPH (iNap sensors) that enable the quantification of cytosolic and mitochondrial NADPH in living cells at high temporal and spatial resolution. Subsequently, Zou et al. established a new technology for simultaneous determination of up to four key redox parameters at single-cell level by using the NADPH probe iNap, NADH probe SoNar and thiol and H_2_O_2_ probes to study changes in redox landscapes during various physiological and pathological processes (Tao et al., 2017; Zou et al., 2018). Zhu et al. established a stable single-neuron intracellular component sampling and mass spectrometry component analysis technology based on electrophysiological patch clamp and electrospray ion source technology, enabling the rapid detection of thousands of small molecules involved in neuronal function, metabolite composition and metabolic pathways in individual neurons in multiple brain regions (Zhu et al., 2017).

There have also been major breakthroughs in the establishment of experimental models for aging studies. In 2018, Yan et al. generated the first knock-in pig model of HD with endogenous expression of full-length mutant HTT that recapitulates disease-related features (Yan et al., 2018). Liao et al. established a new adult heart injury and regeneration model using diploid Xenopus tropicalis (Liao et al., 2017). Mao et al. developed a 2BCas strategy-based Xenopus tropicalis gene mapping technology, providing a useful tool for aging and regeneration research (Mao et al., 2018). Using the cancer transcriptome data from the TCGA database, Xu et al. constructed a tag for single-gene mutations for human coding genes and developed a drug relocation method, having discovered seven drugs potentially prolonging lifespan (Xu et al., 2018a). Yan et al. generated genetically enhanced human vascular cells by targeting a single longevity gene, FOXO3, by gene editing technology, revealing a new mechanism for the long-lived protein FOXO3 in maintaining human vascular homeostasis and opening up the possibilities of generating high-quality safe human cell therapeutic materials with dual resistance to cellular senescence and tumorigenesis (Yan et al., 2019).

With the advancement of sequencing technology, bioinformatics plays an increasingly more important role in aging research. The integration of multi-omics data facilitates the understanding of aging process at the system level. Cheng et al. found that K3K27ac modification plays distinct regulatory roles during aging through integration analysis of human and mouse brain epigenetic and gene expression data. In particular, H3K27ac modifications are enriched in immune-related genes that are upregulated with age and these modifications are gradually lost over time (Cheng et al., 2018). Liu et al. reported the dynamic changes by RNA-seq and cap analysis of gene expression and sequencing (CAGE-seq) in developmental and aging macaque brains and found that the expression of brain-specific lncRNAs are of high region-, gender-, and age-specificity (Liu et al., 2017). Mendez-Bermudez et al. employed ChIP-seq with molecular comb technology and atomic force microscopy and found that the telomere-specific protective protein TRF2 plays an important role in the maintenance of heterochromatin regions, establishing a direct link between telomere and heterochromatin replication (Mendez-Bermudez et al., 2018). The deep mining of high-throughput data also provides new opportunities for aging research. Zhang et al. found that the longevity protein SIRT6 regulates the homeostatic balance of stem cell proliferation and differentiation as well as the development of primates (Pan et al., 2016; Zhang et al. 2018b).

Aging leads to organ degeneration, which may progress into age-related diseases. The plasticity (or reversibility) between organ aging and degeneration provides a critical therapeutic window for the prevention and treatment of age-related diseases. Despite the enormous challenges in aging research, China has launched a broad and comprehensive research program aimed at discovering new driving factors of organ degeneration, delaying the onset and progression of age-related diseases, thus achieving healthy aging. With the emergence of new techniques such as gene-editing, single-cell sequencing, bioinformatics, super-resolution microscopy, metabolite and protein mass spectrometry, and non-human primate modeling, aging research will be greatly facilitated and advanced both in and outside China.

The development of second-generation sequencing technology has enabled transcriptomic and epigenomic analysis at the single-cell level. Single-cell RNA sequencing (scRNA seq) refers to the data mining of genome-wide gene regulatory networks in individual cells. Single-cell sequencing technology was initially used for the identification and classification of highly heterogeneous stem cells and cell populations obtained from early embryonic development (Bao et al., 2009; Tang et al., 2009, 2010), as well as for the analysis of routes and related gene regulatory networks during cell differentiation, reprogramming and transdifferentiation (Macosko et al., 2015; Shekhar et al., 2016; Han et al., 2018; Spanjaard et al., 2018). The key of single-cell sequencing is to individually analyze cells of heterogeneity in any given tissue, thus providing valuable information at the single-cell level for the reconstruction of the entire system. For most mammalian tissues, genetic heterogeneity increases with age and independent of genetic background (Liu et al., 2005; López-Otín et al., 2016; Sen et al., 2016). Cell-to-cell transcriptional variability is often higher in older animals and thus a hallmark of aging (Campisi, 2005; Kirkwood, 2005; López-Otín et al., 2013). Single-cell sequencing technology not only helps us understand the multi-omic differences between individual cells (Davie et al., 2018; Hammond et al., 2018), but also provides a useful tool to precisely classify cells of different subtypes (Enge et al., 2017). Various mutations that accumulate during the process of cell division and the transfer of mobile genetic elements over time are often causatively linked with age-related diseases (López-Otín et al., 2013). Single-cell technology enables the study of cell-to-cell transcriptional variability during aging, providing important clues for the identification of novel driving factors of aging.

Non-human primates are physiologically closer to humans compared to many other species and are thus one of the most ideal *in vivo* models for the study of human organ aging and degeneration (Nelson and Winslow, 2009; Vallender and Miller, 2013; Phillips et al., 2014). At the same time, human organoid models are important achievements in the field of stem cell research. Human stem cell-based micro-organs are produced *in vitro* and used for the study of development, aging and degeneration as well as diseases in 3D dimension (Fatehullah et al., 2016; Quadrato et al., 2016; Pas, 2018; Wimmer et al., 2019). In combination with gene-editing for aging and disease modeling, human organoids are of great theoretical and practical value to mechanistic studies, drug screening, and drug sensitivity and resistance tests.

Since its debut as a gene-editing tool in 2012, CRISPR/Cas9 has been widely used to tinker with genomes of many organisms. Compared with the ZFN and TALEN, CRISPR/Cas9 is simpler, cheaper, quicker and more efficient, although its off-target effect is higher due to the shorter guide RNA sequence compared to those for ZFN or TALEN (Wang et al., 2016b; Adli, 2018). Nevertheless, CRISPR/Cas9-mediated gene-editing has expanded the potential use of stem cells for clinical diagnosis and treatment against aging and related-diseases. Besides the aforementioned gene-editing tools, helper-dependent adenoviral vectors (HDAdVs) is another promising and relatively safe tool for gene integration via homologous recombination (HR) to avoid random integration of between exogenous and chromosomal DNA(Yu et al., 2014). With this tool, Yang et al. and Yan et al. obtained genetically enhanced human stem cells with dual resistance to cellular senescence and tumorigenic transformation, providing possibly better materials for stem cell therapy (Yang et al., 2017; Yan et al., 2019).

The association between organ aging and multiple factors such as chronic inflammation, metabolic imbalance, subcellular organelle dysfunction (e.g., endoplasmic reticulum stress, mitochondrial dysfunction) has received increasingly more attention in recent years (Franceschi and Campisi, 2014; Liu et al., 2014b; Guo et al., 2017). Accordingly, new techniques are applied not only to the mechanistic study of aging but also to aging prediction, examplified by the use of mitoflash frequency to predict the lifespan of nematodes and of artificial intelligence (AI) to recognize human face and predict aging (Shen et al., 2014; Chen et al., 2015).

In China, tremendous efforts are being made to advance comprehensive aging research. A variety of innovative biomedical strategies have facilitated the mechanistic studies of organ aging and degeneration. It is of scientific and clinical significance to understand human aging, search for new intervention targets, develop geroprotective drugs, and achieve human “healthy aging”. It is also important to continue to develop innovative tools and advance in emerging fields such as epigenomics and system biology. Additional efforts need to be invested into basic aging research, healthcare education, public awareness, and life quality improvement of the elderly population, so as to proactively respond to social, economic, and medical problems in an aging society. Beyond scientific efforts, Chinese researchers will also keep focusing on ethical issues in aging research and actively promote the establishment and implementation of ethics laws and regulations.
